# The Impact of Family Functioning Factors on Smartphone Addiction and Phubbing among Muslim Adolescents in Thailand

**DOI:** 10.3390/children11050522

**Published:** 2024-04-26

**Authors:** Yejin Kim, Wanchai Dhammasaccakarn, Kasetchai Laeheem, Idsaratt Rinthaisong

**Affiliations:** 1 Human and Social Development, Faculty of Liberal Arts, Prince of Songkla University, Hat Yai 90110, Thailand; 2 Public Administration, Faculty of Management Sciences, Prince of Songkla University, Hat Yai 90110, Thailand

**Keywords:** family functioning factors, smartphone addiction, phubbing behavior, mediating effect, Muslim adolescents in Thailand, a cross-sectional study

## Abstract

Background: While there is research on protective factors against smartphone addiction (SA) and phubbing, which impact adolescents’ physical, psychological, interpersonal, and academic well-being, focused studies on these issues among Thai Muslim students in Southern Thailand remain scarce. Objectives: To bridge this gap, this research aimed to explore the influence of five family functioning factors—discipline, communication and problem-solving (CPS), relationship, emotional status, and family support—guided by family systems theory and the McMaster Model, on SA and phubbing. Methods: Data from 825 Thai Muslim adolescent secondary school students (Female N = 459 (55.7%), M_age_ = 15.11 ± 1.78) across three southern Thai provinces were analyzed utilizing structural equation modeling (SEM). Results: Significant connections were identified between family functioning factors, particularly emotional status and discipline, and SA, and their impact on phubbing behavior. SA was found to mediate the relationship between these two family factors and phubbing. Interestingly, a higher quality of family relationships was found to be associated with an increased risk of SA, while the other two family functions—CPS and family support—demonstrated no significant association with these digital behaviors. Conclusions: Despite its limitations, including a cross-sectional design and reliance on self-reports, the study underscores the importance of specific family functions in preventing and addressing adolescent SA and phubbing.

## 1. Introduction

In the contemporary digital era, smartphones have transcended their traditional role as communication tools, becoming integral to the daily activities of adolescents worldwide. Their omnipresence, particularly among youth, has led to a seamless integration into various aspects of life, from social interactions to information access [1,2,3]. However, the widespread adoption of smartphones is a double-edged sword. The boundary between beneficial and harmful usage blurs when individuals, especially adolescents, fail to regulate their engagement, potentially impairing their social ties, professional aspirations, and overall well-being [4,5].

The concept of “smartphone addiction” (SA) has emerged in academic discourse to describe a deep-seated dependence on these devices, a phenomenon often linked with problematic smartphone use (PSU) [6]. While SA is not officially classified as a distinct disorder in the DSM-IV, its behavioral symptoms closely resemble the criteria set for other addictive disorders, positioning it similarly to substance abuse behaviors [7,8]. Furthermore, the term “phubbing” emerged to describe a new human behavior characterized by ignoring others in favor of focusing on one’s smartphone, often held in one hand [9]. This behavior reflects how digital devices can disrupt social interactions and relationships. The COVID-19 pandemic significantly increased reliance on smartphones, highlighting their importance in remote learning, maintaining social connections, and entertainment during isolation periods [10].

### 1.1. Impacts of Smartphone Addiction 

This trend was particularly pronounced among adolescent students, for whom smartphones became essential educational tools. Additionally, previous studies have highlighted that those adolescents, who are in critical stages of cognitive, emotional, and social development, are especially susceptible to the immediate rewards provided by smartphones. This vulnerability can adversely affect essential developmental areas, including social competencies, sleep quality, and academic achievements [11,12,13].

Previous research has examined the impacts of smartphone usage on both physical and psychological health, with a particular emphasis on the more profound psychological effects [14,15,16]. Although research on the physical impacts of SA is less prevalent, habitual smartphone use is associated with several physical health challenges. These include “text neck”, eye strain, sleep disturbances, obesity, reduced physical fitness, and pain or migraines due to prolonged sedentary behavior [1,16]. Alshobaili and AlYoousefi [17] specifically linked SA to sleep deprivation.

SA predominantly affects emotional health. Constant notifications and the desire to stay connected can lead to anxiety, while exposure to idealized social media images can result in feelings of inadequacy, depression, and social isolation [18,19]. Previous empirical research has established connections between SA and various mental health issues, including depression and anxiety [20,21,22]. Wu et al. [23] observed that excessive use of smartphones is associated with an increase in depressive symptoms and anxiety, which can negatively affect sleep quality.

The distraction of smartphones can lead to fragmented attention and poor academic performance, with some adolescents resorting to academic dishonesty due to easy access to information [2]. Research by Goswamee and Banerjee [2] and Sahin [24] shows that high smartphone use correlates with decreased academic performance and increased procrastination. Yang, Asbury, and Griffiths [22] observed that university students with significant SA experienced increased academic anxiety and procrastination.

Understanding the long-term effects of smartphone habits is crucial, particularly during adolescence—a key phase in personal development [25]. It is worth noting that not all research views smartphone usage negatively. For instance, Lopez-Fernandez et al. [26] argued that regular mobile gaming does not necessarily lead to SA.

### 1.2. Impacts of Phubbing 

Additionally, this study delves into “phubbing,” a term coined in 2012 combining “phone” and “snubbing”. Phubbing refers to the growing trend of preferring smartphone use over direct interactions with others, signifying a major shift in contemporary communication practices, facilitated by the widespread use of smartphones [9,27,28]. The phenomenon of phubbing poses significant challenges to personal relationships, frequently causing feelings of exclusion and frustration among individuals who feel neglected due to digital distractions [29,30].

The impact of phubbing extends beyond the immediate emotional responses it evokes. Research by Dwyer et al. [31] emphasizes that phubbing substantially degrades the quality of social interactions. It disrupts the flow of conversation and undermines the richness of face-to-face interactions that are fundamental to building and maintaining strong social bonds. Furthermore, a study by Misra et al. [32] revealed that phubbing can adversely affect communication skills and empathetic concern, essential components of healthy and effective interpersonal communication.

Relationship and trust dynamics are also profoundly affected by phubbing. As noted by Przybylski and Weinstein [33] and Cameron and Webster [34], along with Roberts and David [35], the intrusion of smartphones into social interactions diminishes the perceived quality of relationships and trust among individuals. This erosion of trust and relationship quality is particularly concerning, as it can lead to long-term implications for social cohesion and individual well-being.

### 1.3. Smartphone Use in Thailand

In Thailand, smartphone usage reflects global trends, but it is influenced by unique cultural and societal nuances. The country’s rapid urbanization and economic growth coexist with traditional values emphasizing community and family ties [36]. This intersection poses both opportunities and challenges for Thai youth in navigating the digital world [37]. Emerging studies highlight the profound role smartphones play in the daily lives of Thai youth. Smartphones stand out as the favored device among Thai schoolchildren, with PCs not far behind.

In contrast, tablets do not share the same appeal, with infrequent usage patterns [37]. Delving into the specifics, while students typically access PCs or tablets just 1–2 times a week, an impressive 64% turn to their smartphones daily, underlining their indispensable role [38]. Recent research by D’souza and Sharma [38] revealed parity in SA levels between Thai and international students studying in Thailand. In another study, Tangmunkongvorakul et al. [39] noted that an overwhelming 99.1% of university attendees in Northern Thailand owned a smartphone, and nearly half of these students devoted at least five hours daily to their devices.

Despite previous research on the digital behavior among adolescents in Thailand, a significant gap exists regarding the study of the Muslim adolescent population in the southern part—a predominantly Muslim area in a Buddhist-majority country that has experienced unrest. Since 2004, there have been 21,328 incidents, resulting in 7314 deaths and 13,584 injuries [40]. Given Thailand’s diverse cultural landscape, understanding this demographic is essential for a comprehensive analysis. To the researchers’ knowledge, this specific group has not been extensively explored.

### 1.4. Current Study and Proposed Relationship Model

To unravel the intricate dynamics of SA and phubbing, this study adopts a family-centric approach, anchoring its analysis in the family systems theory and the McMaster Model. The family, as a critical social unit in shaping behavioral patterns and belief systems, plays a significant role in the context of SA, as delineated by Bowen’s family systems theory [41,42,43]. This theory emphasizes the dynamics within familial interactions and between the family unit and its encompassing environments, aiming to elucidate the genesis of externalizing problems like SA and phubbing. It posits that such issues are not the result of internal psychological dysfunctions but stem from maladaptive patterns within the family system. Illustratively, concepts such as differentiation of self and the emotional triangle are pivotal in understanding how family dynamics affect behavior [42,43]. Differentiation of self involves an individual’s ability to maintain their autonomy while still being emotionally connected to the family, influencing their resilience to external pressures like the lure of excessive smartphone use.

Supporting this perspective, a study by Kim et al. [25] demonstrates how positive family dynamics, characterized by effective communication and emotional support, can foster self-control in adolescents, a key factor in mitigating the risks of SA. In the context of this study, phubbing is examined as a behavior influenced by both SA and family functioning, with a reciprocal relationship suggesting that while SA and family dynamics can enhance phubbing, phubbing can similarly impact family interactions. This highlights the complexity of addressing phubbing within family and societal contexts, underlining the importance of interventions targeting both digital overuse symptoms and underlying family relationship dynamics to maintain healthy social interactions in the digital era.

To examine how family functions influence SA and phubbing, this study integrates the McMaster Model with family systems theory, offering a detailed analysis of family dynamics and identifying prevalent structures in families facing difficulties [44,45,46]. The model outlines six essential areas of family functionality: problem-solving, communication, roles, affective responsiveness, involvement, and behavior control [45]. In Thailand, this model has been adapted to measure family functionality uniquely, including aspects like emotional status, discipline, relationship, communication and problem-solving (CPS), and family support [46]. However, investigations into how these aspects of family functioning influence SA and phubbing are scarce. Specifically, there has been no research focusing on adolescents within Muslim families, which have unique family systems, in Southern Thailand.

**Figure 1:**
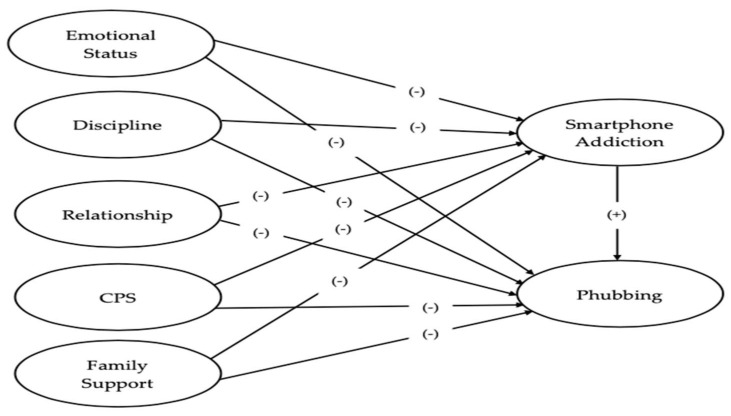
The proposed relationship model of this study. **Note**. CPS = communication and problem-solving.

Therefore, this study aimed to investigate the influence of family functioning aspects on SA and phubbing behaviors among Thai Muslim adolescent students, delving into the intricate relationships between family dynamics, SA, and phubbing among Thai Muslim adolescent students in Southern Thailand. The study proposed a model based on several hypotheses (see Figure 1): that the five factors of family functioning—(1) emotional status, encapsulating the family’s emotional ambiance, manifested through the appropriate expression and range of emotions in reaction to diverse stimuli; (2) discipline, elucidating the family’s proficiency in delineating clear roles, boundaries, and managing member behaviors critical for ensuring physical safety, psychological well-being, and fostering interpersonal socialization; (3) relationship, representing an individual’s subjective appraisal of the efficacy and quality of familial interactions; (4) CPS, focusing on the family’s capacity to effectively address and resolve conflicts through both verbal and non-verbal exchanges, maintaining optimal family functioning; and (5) family support, quantifying the extent to which family units collectively demonstrate interest in, and endorse, the pursuits and well-being of each member, thereby facilitating individual growth and cohesion [44,45,46], as outlined by family systems theory and the McMaster Model—have a negative effect on both SA and phubbing. Furthermore, the study predicted that SA results in increased phubbing behaviors and serves as a mediator between the five aspects of family dynamics and phubbing.

## 2. Materials and Methods

### 2.1. Research Design and Participants

The study employed a cross-sectional research design, allowing for the examination of various variables and their relationships at a single point in time. This approach is particularly useful for identifying patterns and correlations within a specific population, such as the influence of family functioning factors on SA and phubbing among Thai Muslim adolescent students. The sample size was established as 20 times the number of variables in the proposed model.

A total of 825 students from six private Islamic secondary schools across three provinces in Southern Thailand were chosen as the effective sample for the study, using a purposive sampling method. The inclusion criteria were Thai Muslim students in secondary schools, aged 12 to 20 (Grades 7 to 12), who were currently attending school and using a smartphone. These eligible students voluntarily participated in the study by completing a set of paper-based questionnaires.

The majority of the sample was female, constituting 55.7% (n = 459), while males made up 43.3% (n = 422). On average, the participants were 15 years old (M = 15.11, SD = 1.78), with most in the ninth grade (M = 9.35, SD = 1.69). Examining family environments, a significant 77.2% (n = 630) were raised in dual-parent homes, while 16.5% lived in single-parent households, including 3.8% (n = 31) with only a father and 12.7% with only a mother.

Patterns in parental education levels were also observed. Approximately 26.2% (n = 205) of fathers and 20.0% (n = 156) of mothers completed primary school as their highest level of education. Furthermore, a notable 75.8% (n = 663) of fathers and 71% (n = 554) of mothers had secondary school education or below as their highest academic achievement. This data indicates a slight linear pattern in the educational attainment of the participants’ parents. Concerning family income, 67.9% (n = 541) reported monthly earnings below 11,000 Baht. Furthermore, 55.2% (n = 440) reported monthly earnings below 9000 Baht, while 22.1% (n = 168) had incomes exceeding 15,000 Baht.

### 2.2. Statistical Analysis

Data analysis in this study was performed using IBM SPSS 29.0 and AMOS 28.0. The analysis involved several steps. First, descriptive statistics were utilized to evaluate the demographic characteristics of the study subjects and to analyze correlations between variables such as discipline, communication and problem-solving, relationship, emotional status, family support, SA, and phubbing. Second, the normality of the distribution of each item was assessed through skewness and kurtosis. Third, sequential analysis using a split-data strategy was applied by randomly allocating 412 cases for exploratory factor analysis (EFA) and 413 for confirmatory factor analysis (CFA) to verify the scale’s factors. Fourth, confirmatory factor analysis (CFA) was conducted to examine factor loading to verify the proposed relationship model. Fifth, structural equation modeling (SEM) was employed to validate the causal model and analyze the relationships between variables, using fit indices like CMIN/df < 3, CFI and TLI > 0.90, RMSEA < 0.05, and SRMR < 0.05 [47,48]. Lastly, mediation effects among the variables were evaluated using a bootstrapping method with 1000 samples and bias-corrected 95% confidence intervals (CIs).

### 2.3. Research Instruments

#### 2.3.1. Family State and Functioning Assessment Scale in Thai (FSFAS-25) 

The FSFAS-25 [46] integrates components from the Family Assessment Device within the McMaster Model of Family Functioning, the Chulalongkorn Family Inventory, and the Thai Family Functional Scale to thoroughly assess family functioning. This 25-item scale covers five key domains: discipline, communication and problem-solving, relationship, emotional status, and family support. It employs a 4-point Likert scale ranging from 1 (strongly disagree) to 4 (strongly agree), including both positively phrased and reverse-scored negative statements. The total score ranges from 25 to 100, with higher scores denoting more effective family functioning. The scale demonstrates notable reliability, with a Cronbach’s alpha of 0.84 in this study.

**Figure 2:**
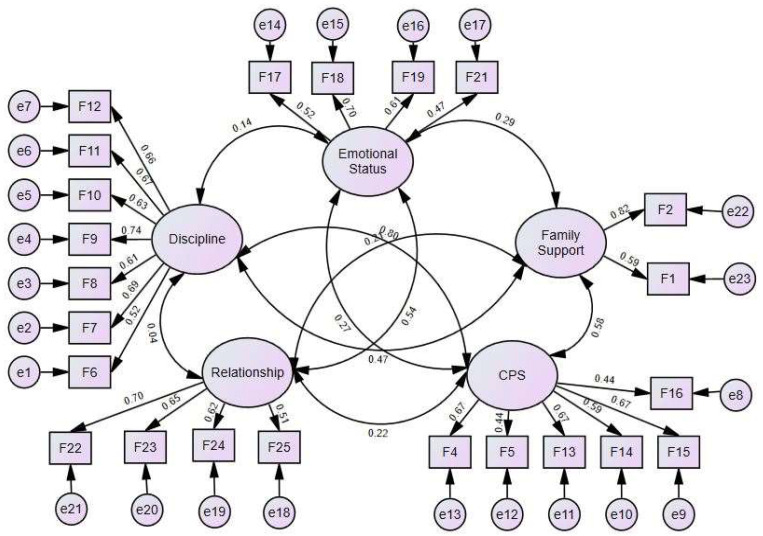
Confirmatory factor analysis for family functioning scale. **Note**. CPS = communication and problem-solving.

The validity of the scale in this study was verified using exploratory factor analysis (EFA) in SPSS, with a Kaiser–Meyer–Olkin (KMO) measure of 0.86 and a significant Bartlett’s Test of Sphericity (*p* < 0.001), confirming accurate factor extraction via principal components with varimax rotation. All five factors, each with an eigenvalue over 1.11, accounted for 51.9% of the variance, reflecting the construct of the original scale: emotional status, discipline, relationship, CPS, and family support. Following subsequent CFA conducted in Amos (χ^2^ = 560.57, DF = 220, CFI = 0.91, TLI = 0.90, RMSEA = 0.05), particularly after removing Item 20 from the emotional status factor and Item 3 from the family support factor due to low factor loading (below 0.40), this structure was supported (see Figure 2). Based on these analyses, the study identified each of these sub-factors as independent or exogenous variables.

#### 2.3.2. Thai Version of Smartphone Addiction Scale–Short Version (THAI-SAS-SV)

In this study, the THAI-SAS-SV, formulated by Hanphitakphon and Thawinchai [49], was used to examine SA. Adapted from Kwon et al.’s [50] smartphone addiction scale–short version (SAS-SV), this tool features specific cut-off scores of 31 for males and 33 for females. It contains 10 items rated on a 6-point Likert scale, from strongly disagree = 1 to strongly agree = 6, with higher scores indicating increased SA. The scale showcased commendable reliability, reflected by a Cronbach’s alpha coefficient of 0.78 in this study.

**Figure 3:**
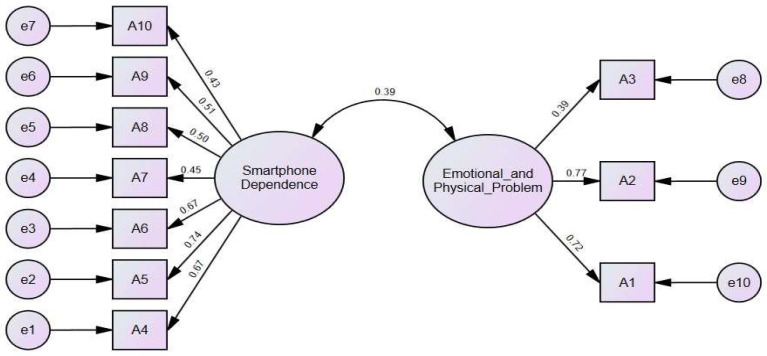
Confirmatory factor analysis for smartphone addiction scale.

The validity of the scale was evaluated through exploratory factor analysis (EFA) conducted in SPSS. The Kaiser–Meyer–Olkin (KMO) measure stood at 0.79, and Bartlett’s Test of Sphericity returned a significant result (*p* < 0.001), confirming the appropriateness of extracting factors using principal components analysis with varimax rotation. The two factors, all with an eigenvalue above 1.51, explained 50.2% of the variance. Factor 1, labeled as “smartphone dependence” by the authors, consisted of 7 items. Factor 2, denoted as “emotional and physical problem”, comprised 3 items. The CFA results (χ^2^ = 117.92, DF = 34, CFI = 0.91, TLI = 0.89, RMSEA = 0.07) validated the scale’s structure, with item loadings for the smartphone dependence sub-factor ranging from 0.43 to 0.74, and for the other sub-factor from 0.39 to 0.77 on a single factor (see Figure 3).

#### 2.3.3. Phubbing

The study employed the revised phubbing scale by Blachnion et al. [51] to assess the level of phubbing behavior among participants. The initial version of this scale by Karadağ et al. [27] featured 10 items, categorized into two subscales: “Communication Disturbance” and “Phone Obsession”. Participants rated each statement on a 5-point Likert scale, spanning from 1 (strongly disagree) to 5 (strongly agree), with the subscales showing good internal consistency (Cronbach’s alpha of 0.87 and 0.85, respectively).

Blachnion and colleagues [51] later revised this scale from 10 to 8 items, removing items 5 and 10 due to inadequate model fit in the original format. This modified 8-item version demonstrated improved model fit across 20 countries and maintained strong internal consistency, with reliability scores ranging from 0.71 to 0.95. Consequently, this research adopts the 8-item revised version for its Thai adaptation.

**Figure 4:**
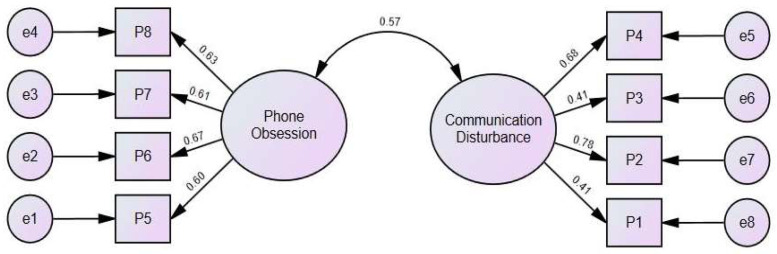
Confirmatory factor analysis for phubbing scale.

The scale’s validity was further evaluated using an EFA. The analysis showed a Kaiser–Meyer–Olkin (KMO) measure of 0.82 and a significant Bartlett’s Test of Sphericity (*p* < 0.001), indicating that the factor extraction was suitable. Principal components analysis with varimax rotation identified two factors, each with an eigenvalue over 1.20. These two factors combined explained 59.0% of the variance, aligning with the scale’s intended constructs: phone obsession and communication disturbance. Additionally, CFA results provided further validation. The CFA metrics (χ^2^= 36.56, DF = 19, CFI = 0.97, TLI = 0.96, RMSEA = 0.04) supported the effective association of items with their respective factors. The analysis further validated that the items loaded effectively, with loadings ranging from 0.61 to 0.67 for the phone obsession sub-factor and from 0.41 to 0.78 for the communication disturbance sub-factor, all aligning with a single, unified factor (see Figure 4).

### 2.4. Data Collection

The research was initiated after securing official permission from six Islamic secondary schools located in three southern provinces of Thailand, selecting two schools per province for participation. In each school, a designated facilitator helped the main researcher by distributing informed consent forms and paper-based questionnaires to students during break times. The study involved students from grade 7 to grade 12.

The main researcher provided informed consent forms outlining the study’s objectives, assurance of response confidentiality, and the voluntary nature of participation. Questionnaires and informed consent forms were distributed to each class voluntarily, following verbal consent from participants. The respondents returned their completed questionnaires along with consent forms signed by themselves and a parent or guardian. Out of the 865 questionnaire sets issued, 15 were not returned. Moreover, 25 responses were disqualified due to inaccurate answers or an absence of parental consent, leaving 825 valid questionnaires with duly signed informed consent forms for inclusion in the final sample. The study was conducted with approval from an Institutional Review Board (IRB).

## 3. Results

### 3.1. Descriptive Statistics and Correlation Analysis 

Among 825 Muslim adolescent participants, a significant portion, 70.30% (n = 580), reported smartphone addiction scores above the established thresholds (over 31 for males and over 33 for females), highlighting a high prevalence of addiction. The average phubbing score among these participants was 22.05, with a standard deviation of 6.43.

Table 1 presents the mean, standard deviation, skewness, kurtosis, and the outcomes of Pearson’s correlation analysis for all the variables studied. The skewness values for each variable ranged between −0.64 and 0.01, while the kurtosis values varied from −0.60 to 0.50. The skewness and kurtosis of each variable were within the normal range (absolute values below 2 and 7, respectively).

In line with our predictions, discipline exhibited a negative and significant correlation with both SA (*r* = −0.09, *p* < 0.05) and phubbing (*r* = −0.08, *p* < 0.05). Similarly, emotional status showed a negative and significant correlation with SA (*r* = −0.21, *p* < 0.01) and phubbing (*r* = −0.20, *p* < 0.01). The factor labeled “relationship” was found to be negatively and significantly correlated with SA (*r* = −0.10, *p* < 0.01), but not with phubbing. Additionally, a positive and significant correlation was identified between SA and phubbing (*r* = 0.38, *p* < 0.01). However, neither CPS nor family support demonstrated significant correlations with SA or phubbing.

### 3.2. Verification of the Relationship Model in the Study

CFA was conducted to evaluate the proposed model, which explored the associations between five family functioning factors, SA, and phubbing. The initial CFA results indicated a reasonable fit: χ^2^/df = 982.12/303 = 3.24, CFI = 0.88, TLI = 0.86, RMSEA = 0.05, and SRMR = 0.05. However, two factors—CPS and family support—were excluded due to their lack of significant correlations with, and predictive power for, SA and phubbing.

Subsequently, the model was adjusted to focus on the remaining three family functioning factors and their relationships with SA and phubbing. The revised model showed an acceptable fit, as demonstrated by the fit indices: χ^2^/df = 484.52/142 = 3.41, CFI = 0.90, TLI = 0.88, RMSEA = 0.05, and SRMR = 0.05. The modification indices suggested adding two covariance pathways to improve the model’s fit: one between the error terms of items 10 and 11 within the discipline factor, and another between items 17 and 18 of the emotional status factors. These adjustments led to a significantly improved fit in the re-specified model, with indices showing χ^2^/df = 401.34/140 = 2.87, CFI = 0.93, TLI = 0.91, RMSEA = 0.04, and SRMR = 0.05. Consequently, this re-specified model, accounting for 3% and 16% of the variance in SA and phubbing, respectively, was selected as the final relationship model.

### 3.3. Path Coefficients of the Final Relationship Model in this Study

**Figure 5:**
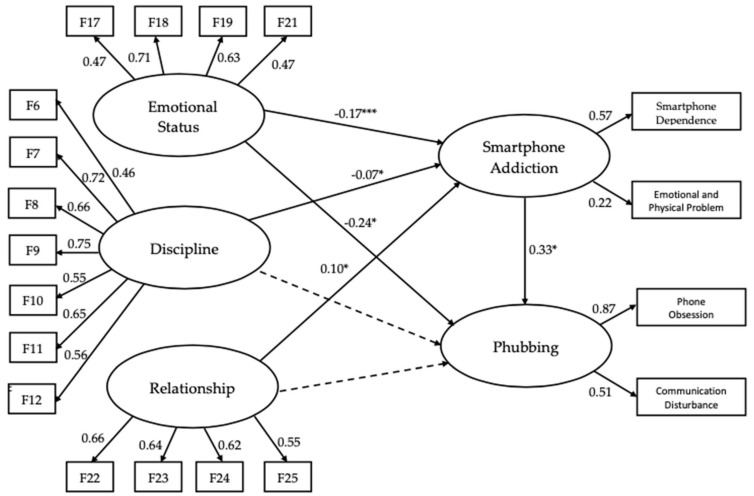
The final relationship model of this study. **Note**. Standardized path coefficients are depicted, with solid arrows representing significant relationships and dotted arrows indicating non-significant relationships. Error terms and *p*-values for paths from observed variables to latent variables are included but omitted in the figure for simplicity. * *p* < 0.05, *** *p* < 0.001.

Figure 5 displays the path coefficients from the SEM analysis, which evaluated the relationships in the final relationship model. The results showed that emotional status had a significant negative effect on both SA (*β* = −0.17, *p* < 0.001) and phubbing (*β* = −0.24, *p* < 0.01). Discipline also demonstrated a significant negative effect on SA (*β* = −0.07, *p* < 0.01), but it did not significantly affect phubbing. In contrast, the factor, relationship, had a positive effect on SA (*β* = 0.09, *p* < 0.05), but its impact on phubbing was not significant. Additionally, SA positively influenced phubbing (*β* = 0.33, *p* < 0.05).

### 3.4. Mediating Roles of SA

The study conducted a mediation analysis to explore how SA acts as a mediator in the relationships between each of the three family functioning factors and phubbing behaviors (see Table 2).

The findings demonstrated that discipline significantly and negatively predicted SA, which subsequently had a positive influence on phubbing (*B* = −0.26, *p* = 0.004, 95% CI [−0.595, −0.045]). In a similar vein, SA was found to be a significant and partial mediator in the relationship between emotional status and phubbing (*B* = −0.48, *p* = 0.003, 95% CI [−0.070, 0.003]). However, the indirect effects of the relationship variable on phubbing through SA were not found to be significant.

## 4. Discussion

This study explored the impact of different family functioning factors on digital behaviors, specifically SA and phubbing, that could adversely affect the physical, psychological, interpersonal, and academic well-being of Muslim adolescent students in Southern Thailand. Since 2004, the region has been under martial law, and its predominantly Muslim population, with distinct family structures and values, such as the social and religious acceptance of polygamy, represents a marginalized group in a Buddhist-majority country. Furthermore, the study reveals that many adolescents’ parents have only achieved a secondary level of education or less, with the majority of families (67.9%) earning a monthly income below 11,000 Baht. This reflects the broader socio-economic challenges faced by this demographic, especially considering that Southern Thailand is subject to the lowest minimum wage mandates in the country.

Given the backdrop of lower socio-economic conditions and distinct family values, these students might be at a heightened risk of SA and phubbing. This is particularly relevant considering prior findings that indicate adolescents from supportive family environments are four times less likely to develop internet addiction compared to those from less supportive backgrounds [52].

The study highlights a significant occurrence of SA among Thai Muslim adolescents, with an alarming 70% exhibiting addiction symptoms. This rate notably diverges from those observed in other Asian contexts. For context, addiction rates in India fluctuate between 39% and 67% [2,53], while only 21.3% of Chinese undergraduates [54], 11.4% of Indonesian junior high students [55], 35.2% of South Korean adolescents [56], and 62.6% of Filipino adolescents [57] are reported as addicted. Interestingly, even within Thailand, there is a stark contrast in SA rates, as evidenced by a recent study by Chinwong et al. [37], which found a 49% addiction rate among undergraduates in Northern Thailand. These disparities necessitate a deeper exploration to understand the unique factors contributing to the high addiction rates among Thai Muslim students.

Drawing on family systems theory and the McMaster Model, this study aimed to assess the effects of five critical family functioning factors on SA and phubbing among Thai Muslim adolescents, employing SEM for analysis. The model, informed by these theoretical frameworks, was rigorously tested and produced significant insights.

The results underscore a vital link between family functioning—specifically, emotional status and discipline—and SA in this group, with emotional status inversely related to phubbing behavior. According to Bowen’s family systems theory, the emotional dynamics within a family significantly affect adolescents’ vulnerability to external challenges like SA [41,42,43]. Teens from environments marked by high emotional intensity, lack of emotional connection, or blurred family boundaries might turn to smartphones as solace for familial emotional discrepancies or confusion. Supporting this notion, research from South Asia indicates that adolescents from families with healthier dynamics are markedly less prone to developing problematic smartphone usage [52]. This indicates that smartphone reliance can often act as a coping strategy for navigating unaddressed emotional challenges within the family structure.

The role of parents emerges as crucial in this dynamic. An adaptable and understanding parent–teen relationship can act as a strong buffer against the allure of smartphones [25,44,45]. Especially during the transformative high school years, such a relationship can significantly influence adolescents’ smartphone habits. A family environment that balances emotional support with clear boundaries can effectively prevent excessive smartphone use among teenagers. This finding aligns with research from Korea and China, emphasizing the diminishing effect of cohesive family environments on SA [58,59]. Mangialavori et al. [60] further highlight family challenges like disengagement and chaos as significant risk factors for SA, underscoring the importance of healthy family interaction patterns.

Moreover, the study reveals that emotional status and discipline within the family context are associated with phubbing behavior, mediated through SA levels. A functional emotional environment and disciplined family setting can reduce the levels of SA, subsequently influencing phubbing behavior. An increase in phubbing among adolescents can adversely impact family dynamics, fostering a sense of neglect among family members and potentially leading to a cycle of family dysfunction [29,61,62].

Interestingly, the study also observes that a higher quality of family relationships paradoxically increases the risk of SA among Thai Muslim adolescents. This counterintuitive finding, where SA acts as a partial mediator between family relationships and phubbing, aligns with observations made by Castaño-Pulgarín et al. [62]. It implies that, paradoxically, stronger family relationships can sometimes inadvertently elevate the risk of addiction. This counterintuitive effect may stem from the shifting social dynamics of adolescence, wherein peer influence and digital engagement begin to overshadow direct family interactions. As a result, adolescents may be drawn into online risks due to diminished discipline and blurred boundaries within the family context [63]. For some young adults, smartphone dependency may also serve as a coping strategy against turbulent family environments [60,63,64]. Additionally, this study found no significant link between CPS and family support with SA or phubbing, suggesting that while family plays a critical role, adolescents are influenced by broader social environments. Employing Bronfenbrenner’s ecological systems theory [65], it underscores the multifaceted social contexts impacting adolescent development, spanning the microsystem (family, peers, school, religion, workplace), mesosystem (interrelations among microsystems), exosystem (industry, media, political landscape, neighborhood, social services), to the macrosystem (societal beliefs and norms). This framework suggests that peer groups, alongside parental dynamics, significantly shape adolescents’ digital habits [66], highlighting the broad spectrum of social influences that contribute to their online behaviors.

This complex relationship underscores the dual role of smartphones in the lives of adolescents: they can be both a symptom of and a solution to family disconnection. Smartphones can offer an escape and a sense of relief from familial stress and anxiety [60,61], yet they might also detract from in-person interactions, challenging the maintenance of strong family ties. Despite smartphones’ capacity to connect families, many individuals still tend to favor virtual interactions over spending physical, quality time with family to build social ties [63,66].

This dynamic highlights the need for a balanced approach in family functioning, where time spent together and adaptability play protective roles against SA among adolescents [58]. This balance is essential in navigating the challenges posed by digital technology in the modern family setting. Addressing SA and phubbing requires concerted efforts from policymakers, educators, and families to strengthen family dynamics, enhance digital literacy, and maintain open parent–child communication. Workshops and educational programs that discuss the risks of excessive smartphone use and prevention strategies are crucial. This strategy is especially relevant in distinct cultural and socio-political environments like Southern Thailand, where the interplay of traditional values and modern-day challenges underscores the importance of customized interventions for managing young people’s digital behaviors. While this study primarily examines Muslim adolescents in Southern Thailand, its insights apply to similar socio-economic and religious contexts in other regions, such as Malaysia, Indonesia, Brunei, and the Philippines.

### Limitations of This Study and Future Research

This research has several limitations. First, this study employed a cross-sectional design, which offers only a snapshot of the phenomenon under investigation. Subsequent studies should consider implementing longitudinal or experimental designs to establish causality and further validate the observed relationships. Second, the research relied on self-reported questionnaires, which can be prone to biases and may not always capture the true depth or breadth of a subject. Future studies should adopt a multi-level approach that includes perspectives from parents, peers, and teachers, alongside a variety of data collection methods such as observations, interviews, and physiological measurements. Third, the assessments of family functioning were based solely on adolescents’ self-reports, potentially providing a one-dimensional perspective. A more holistic approach would entail sourcing data from various family members, such as parents and siblings, to provide a multi-faceted understanding. This could also involve the use of additional tools like dysfunctional family measurements. Fourthly, the study was limited to Muslim adolescent students in Southern Thailand. Generalizing findings to broader religious populations should be done cautiously. Future research should include a more diverse sample to understand nuances across different religious groups. Lastly, the study presented conflicting results, where some family functioning factors were predictive of SA while others, like family relationships, positively influenced addiction tendencies. Subsequent research should aim to replicate these findings and explore the underlying reasons for such discrepancies.

## 5. Conclusions

The insights of this study into the intricate dynamics between family functions, SA, and phubbing among Thai Muslim adolescents underline the necessity of enhancing self-regulation skills, particularly during the critical period of transitioning from childhood to adulthood. The results indicate that nurturing a positive family emotional climate and maintaining disciplined practices are crucial in reducing the risks related to SA and phubbing.

## Figures and Tables

**Table 1:** Pearson’s correlations among the variables.

Variables	1	2	3	4	5	6	7
1. Discipline	-	0.59 **	0.03	0.10 **	0.38 **	−0.09 *	−0.08 *
2. CPS		-	0.16 **	0.17 **	0.48 **	0.01	−0.07
3. Relationship			-	0.40 **	0.17 **	−0.03	−0.10 **
4. Emotional status				-	0.20 **	−0.21 **	−0.20 **
5. Family support					-	0.02	−0.06
6. Smartphone addiction						-	0.38 **
7. Phubbing							-
Mean	18.69	17.18	12.69	10.50	5.82	36.82	22.05
Standard deviation	4.72	3.69	2.79	2.74	1.52	8.41	6.43
Skewness	−0.09	−0.22	−0.64	−0.12	−0.34	−0.18	0.01
Kurtosis	−0.09	−0.38	−0.33	−0.52	−0.60	−0.27	0.05
Cronbach’s α	8.2	0.74	0.71	65	0.63	0.78	0.78

**Note**. CPS = communication and problem-solving; ** *p* < 0.01; * *p* < 0.05.

**Table 2:** The mediation effects of smartphone addiction using 1000 bootstrap samples.

Indirect Effect Paths	Estimate	*p*	95% Bias-Corrected CI	
			Lower	Upper
Discipline ⟶ SA ⟶ Phubbing	−0.26	0.004	−0.595	−0.045
Relationship ⟶ SA ⟶ Phubbing	0.17	0.064	−0.005	0.585
Emotional Status ⟶ SA ⟶ Phubbing	−0.48	0.003	−0.070	0.003

## Data Availability

Data availability is restricted due to privacy reasons. However, data may be available by writing to the correspondence author.
